# Physical Activity and Cognitive Function Among Chinese Adults Aged 45 Years and Older with Self‐Reported Hearing Loss: A 9‐Year Longitudinal Study

**DOI:** 10.1002/brb3.71646

**Published:** 2026-08-20

**Authors:** Xiaoyang Li, Yingxin Tang, Ruxia Qiu, Chi Zhang, Yinan Zhao, Hui Feng, Xiaoling Zhang

**Affiliations:** ^1^ Xiangya School of Nursing Central South University Changsha China; ^2^ Nursing Department The Central Hospital of Xiangtan (The Affiliated Hospital of Hunan University) Xiangtan China

**Keywords:** cognition, exercise, group‐based trajectory modelling, hearing loss, longitudinal studies

## Abstract

**Background:**

Physical activity may help prevent dementia in people with hearing loss, but evidence on long‐term behavioural heterogeneity is limited. This study examined dose–response associations between physical activity and cognitive impairment and explored heterogeneity in long‐term physical activity trajectories and their associations with cognitive function among adults with self‐reported hearing loss.

**Methods:**

Data were from the China Health and Retirement Longitudinal Study (2011–2020), including 2055 adults aged ≥ 45 years with hearing difficulties and normal cognition at baseline. Physical activity, assessed biennially using the International Physical Activity Questionnaire, was expressed as metabolic equivalent minutes per week. Cognitive function in 2020 was measured by validated tests of episodic memory and mental status (range 0–21). Multiple linear regression examined associations between activity trajectories and cognition.

**Results:**

Physical activity showed an L‐shaped relationship with cognitive impairment. Three trajectories were identified: slight decline (70.61%), sharp drop (15.28%), and rise‐then‐fall (14.11%). Compared with the slight‐decline group, the rise‐then‐fall group had lower global cognition (*β* = −0.621, 95% CI −1.022–−0.219) and mental status scores (*β* = −0.449, 95% CI –0.648–−0.249). This trajectory was more common among men, younger rural residents, frequent drinkers, non‐nappers, and those with depressive symptoms.

**Conclusions:**

Among adults with self‐reported hearing loss, physical activity showed an L‐shaped association with cognitive impairment, and participants following an unstable rise‐then‐fall activity pattern had lower cognitive function. These findings highlight heterogeneity in physical activity patterns within this population and support the need for strategies that help sustain long‐term physical activity engagement.

**Trial registration:**

Not applicable.

## Graphical abstract

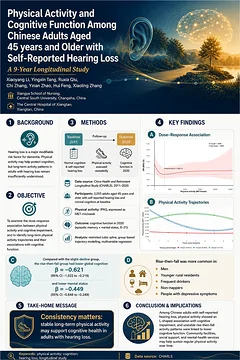

**Abbreviations**
CHARLS: China Health and Retirement Longitudinal Study
CI: confidence interval
GBTM: group‐based trajectory modelling
IPTW: inverse‐probability‐of‐treatment weighting
RCS: restricted cubic spline
STROBE: strengthening the reporting of observational studies in epidemiology

## 1 Background

Dementia, a persistent and severe form of cognitive impairment, has become a major global health challenge (2024 Alzheimer's disease facts and figures). In China, its prevalence among adults aged 65 years and older is 9.1% and is projected to increase as the population ages (Gan et al. 2024). Dementia often co‐occurs with comorbidities such as cardiovascular disease, adding strain to healthcare systems and diminishing quality of life (Li et al. 2025). As no curative treatment exists (Lee et al. 2022), prevention has become a priority.

Hearing loss in middle‐aged and older adults is a leading, modifiable risk factor for dementia and a risk factor for social withdrawal and depression (Livingston et al. 2020; Gallagher et al. 2024; Xu et al. 2023). In China, an estimated 115 million people had moderate‐to‐complete hearing loss in 2015, accounting for 8.4% of the total population; among them, 85.7% were older than 50 years, corresponding to approximately 99 million people (Wang et al. 2025). Therefore, preventing dementia in populations with hearing loss has attracted increasing attention. Although hearing aids are considered the optimal intervention for reducing hearing‐related cognitive decline, their adoption remains low due to barriers in accessibility, affordability, and stigma (Lin 2024).

Regular physical activity has been shown to reduce the risk of cognitive decline in older adults (Iso‐Markku et al. 2024; Venegas‐Sanabria et al. 2022; Domingos et al. 2021). Additionally, a composite healthy lifestyle, including regular physical activity, is associated with a lower risk of cognitive impairment among individuals with hearing loss when compared with hearing‐normal individuals (Zhao et al. 2024). Experimental studies further confirmed that physical activity can alleviate the cognitive improvement in mice with hearing loss. In particular, physical activity enhances cognition by mitigating hippocampal neurogenesis decline and microglial dysfunction (Zhuang et al. 2023). Therefore, physical activity may contribute to the preservation of cognitive health in the aging population with hearing loss.

However, mixed results exist when delineating the dose‒response relationship between physical activity and cognitive performance. One meta‐analysis revealed a linear trend (Xu et al. 2017); two meta‐analyses reported a nonlinear relationship, with the threshold for minimal/maximum benefit varying (Iso‐Markku et al. 2024; Gallardo‐Gómez et al. 2022). Moreover, these studies only examined the relationship from the perspective of average physical activity levels (cross‐sectional or mean estimates) (Matthews et al. 2024). Given the greater heterogeneity in activity among people with hearing loss than among those with normal hearing (Goodwin et al. 2023), the average physical activity levels are inadequate to capture temporal variability and underlying patterns. Therefore, the optimal thresholds of physical activity in this population remain elusive, let alone those in the Chinese population, underscoring the need to examine long‐term trajectories.

Therefore, this study aims to examine the association between physical activity and cognitive function within Chinese middle‐aged and older adults with self‐reported hearing loss, using data from the China Health and Retirement Longitudinal Study (CHARLS) ranging from 2011–2020: using 2020 cross‐sectional data to determine the threshold of physical activity; using longitudinal data to determine the heterogeneity of physical activity patterns—determined by group‐based trajectory modelling—and their associations with cognitive function, thereby describing the associations between physical activity patterns and cognitive function in this population and facilitating more refined strategies to support cognitive health in older adults with hearing loss.

## 2 Methods

### 2.1 Study Participants

The CHARLS is a nationwide prospective cohort of community‐dwelling adults aged ≥ 45 years that is used to assess their social, economic, and health conditions. This study used longitudinal data from the 2011, 2013, 2015, 2018, and 2020 waves. All the surveys were approved by the Biomedical Ethics Review Committee of Peking University (IRB00001052‐1015). All procedures were conducted in accordance with the ethical principles outlined in the Declaration of Helsinki (2013 revision) and relevant institutional guidelines and regulations. All participants were informed about the purpose of the survey, assured of confidentiality, and provided written informed consent prior to participation. Participation was voluntary and could be withdrawn at any time without consequence (Zhao et al. 2014). As this study was a secondary analysis of anonymized publicly available CHARLS data, no additional ethical approval was required. As this was not a clinical trial, a clinical trial registration number was not applicable. The study followed the STROBE reporting guidelines (Table S1 in the Supporting Information) (Vandenbroucke et al. 2014).

As the objective of this study was to characterize heterogeneity in long‐term physical activity patterns within adults with self‐reported hearing loss, participants without self‐reported hearing loss were not included in the primary analyses. Participants were included if they: (1) were aged ≥ 45 years; (2) reported hearing loss at baseline (those rating their hearing as fair or poor in response to “Is your hearing excellent, very good, good, fair, or poor?” were classified as having hearing loss); and (3) had at least three assessments of physical activity, including data from both 2011 (baseline) and 2020 (Wave 5). The exclusion criteria were as follows: (1) cognitive impairment at baseline; (2) absence of cognitive assessment data in Wave 5; and (3) loss to follow‐up or death. In total, 2055 participants were included (Figure S1; Table S2).

### 2.2 Physical Activity Measurement (Based on Wave 1–5 Data)

Physical activity, including intensity and duration, was measured by the International Physical Activity Questionnaire across Waves 1–5 of the survey (CRAIG et al. 2003). Intensity was classified as vigorous, moderate, or light, and duration was categorized as < 30 min, 0.5–2 h, 2–4 h, and > 4 h. Because CHARLS collected physical activity duration using categorical rather than continuous responses, we converted each duration category into an approximate daily duration to calculate MET‐min/week. Specifically, the first three categories were assigned their midpoint values, and the open‐ended highest category (> 4 h) was assigned 4 h. This approach was used to provide a conservative estimate of physical activity volume and to reduce the influence of extreme values in the open‐ended category. Total weekly physical activity was then calculated as MET‐min/week: (8.0 × minutes of vigorous activity × days/week) + (4.0 × minutes of moderate activity × days/week) + (3.3 × minutes of walking × days/week) (Hu et al. 2022).

### 2.3 Cognitive Function Measurement (Based on Wave 5 Data)

Cognitive function, as assessed in Wave 5, was measured in two dimensions: episodic memory and mental status. These cognitive assessments were administered as part of the standardized CHARLS interview by trained interviewers. Episodic memory was evaluated via word recall tasks: immediate word recall (range: 0–10) and delayed word recall (range: 0–10), with the final score calculated as the average of the two. Mental status (0–11 points) was assessed on the basis of items from the telephone interview for cognitive status, including orientation (reporting the current month, day, year, season, and weekday; 0–5 points), attention (serial subtraction of 7 from 100, repeated five times; 0–5 points), and visual construction (redrawing a previously shown figure; 0–1 point). The total score of cognitive function was the sum of these 2 dimensions (range: 0–21), with higher scores indicating better cognitive function. In the present study, cognitive impairment was defined as a total score of < 6 (Jak et al. 2009; Li et al. 2022).

### 2.4 Covariates (Based on Wave 5 Data)

The covariates included sociodemographic characteristics, lifestyle factors, and health‐related conditions previously associated with cognitive impairment, all of which were measured in Wave 5. Sociodemographic variables included sex, age, place of residence, education level, and marital status, as these factors are closely linked to cognitive reserve (Subramaniapillai et al. 2021; Muhammad 2023; Arora et al. 2024; Liu et al. 2019). Lifestyle factors included smoking status, alcohol consumption, nighttime sleep duration, and daytime napping duration on the basis of prior evidence linking these behaviors to neurocognitive outcomes (Bloomberg et al. 2024; Zijlmans et al. 2022; Duan et al. 2023; Leng et al. 2019; Lin et al. 2018).

Health‐related conditions included chronic disease status and depressive symptoms. Chronic disease status was determined by self‐reports (diagnosis confirmed by physicians), including but not limited to hypertension, dyslipidemia, diabetes, cancer, chronic lung disease, heart disease, stroke, liver disease, kidney disease, gastrointestinal disease, emotional or mental health conditions, memory‐related disorders (Alzheimer's disease, brain atrophy), Parkinson's disease, and arthritis or rheumatism. In this study, we further categorized them according to the number of chronic conditions (none, 1–2, or ≥ 3) (Xu et al. 2020). Depressive symptoms (never diagnosed by physicians) were assessed by the 10‐item version of the Center for Epidemiologic Studies Depression Scale, with a cut‐off ≥ 12 indicating clinically significant depression (Forbes et al. 2024; Li et al. 2023). Because cognitive function was assessed in Wave 5, Wave 5 covariates were used to account for participants’ sociodemographic, behavioral, and health‐related characteristics at the time of cognitive assessment, rather than their baseline status nine years earlier.

### 2.5 Statistical Analysis

The analytical strategies addressed complementary research questions: physical activity quintiles and restricted cubic spline (RCS) models were used to examine the cross‐sectional dose–response association in Wave 5, whereas GBTM was used to characterize longitudinal heterogeneity in physical activity patterns across Waves 1–5 and their associations with cognitive function in Wave 5. Accordingly, the results section is presented in the same sequence: Wave 5 dose–response analyses, trajectory identification, descriptive trajectory characteristics, and trajectory–cognition associations.

Categorical variables are presented as frequencies and percentages, and chi‐square tests were used to examine differences between groups. When overall tests were significant, post hoc pairwise comparisons with Bonferroni correction were performed. Continuous cognitive scores, including total cognitive function, episodic memory, and mental status, were presented as medians and interquartile ranges because of their skewed distributions, and between‐group comparisons were performed using the Kruskal–Wallis H test. When overall tests were significant, post hoc pairwise comparisons with Bonferroni correction were performed.

Multivariable logistic regression was used for three purposes: (1) to explore the cross‐sectional associations between physical activity quintiles and cognitive impairment in Wave 5, with physical activity in Wave 5 categorized into quintiles (Q1–Q5) according to its distribution among the analytic sample (Celis‐Morales et al. 2017), given the nature of potential nonlinear associations; (2) to assess the associations between physical activity trajectory and binary cognitive impairment in Wave 5; and (3) to explore the potential influencing factors of trajectories.

RCS models were used to flexibly assess the potential nonlinear dose–response association between physical activity and cognitive impairment in Wave 5. This approach was selected because the association between physical activity and cognitive outcomes may be nonlinear and may involve a threshold effect. Physical activity in Wave 5 was modelled as a continuous variable using five knots, with knot locations automatically placed at the default quantiles of the physical activity distribution using the rms package in R. The threshold was identified from the fitted RCS curve as the physical activity level corresponding to the lowest predicted odds of cognitive impairment. “High physical activity exposure” was defined as activity ≥ the RCS‐derived threshold in at least three waves, Waves 1–4.

The physical activity trajectories across Waves 1–5 (2011–2020) were identified via group‐based trajectory modelling (GBTM) (hlme(), lcmm, R). Details of the model selection process are provided in the Supporting Information. Because physical activity in Wave 5 contributed to trajectory classification and cognitive function was assessed in the same wave, the trajectory–cognition analysis does not represent a strictly prospective exposure–outcome relationship. Rather, trajectory membership reflects long‐term patterns of physical activity up to the time of cognitive assessment.

In addition to analyses using binary cognitive impairment, analyses using continuous cognitive scores were specified as part of the analytic plan to preserve information across the full cognitive score distribution. Multivariate linear regression was applied to assess associations with continuous cognitive function scores in Wave 5, including total cognitive function, episodic memory, and mental status, to show cognitive differences across physical activity trajectories, and to examine the interaction of trajectories and high physical activity exposure.

Sensitivity analyses were conducted to assess the robustness of the results (see the Supporting Information for details). All the statistical analyses were conducted with R software (version 4.3.2) and Stata (version 18.0), and a two‐sided *p* < 0.05 was considered statistically significant. Data analyses were conducted from May to August 2025.

## 3 Results

### 3.1 Demographic Characteristics of the Participants

A total of 2055 participants were included (mean age = 57.8 ± 7.9 years; 46.7% male; Table S2). Among them, 281 participants (13.67%) had cognitive impairment. The participants with cognitive impairment tended to be older, single, rural‐dwelling, have lower levels of education and alcohol consumption, report longer sleep and napping, experience more depressive symptoms and poorer baseline hearing, and have lower baseline total cognitive function, episodic memory, and mental state scores (all *p* < 0.05).

### 3.2 Cross‐Sectional Association between Physical Activity and Cognitive Impairment in Wave 5

In the cross‐sectional dose–response analysis based on Wave 5 data, physical activity was categorized into quintiles according to its distribution in the analytic sample. After adjusting for sociodemographic, lifestyle, chronic conditions, and depression status, only the risk for cognitive impairment in Q3 significantly decreased by 52% compared with that in the lowest quintile group (Q1) (adjusted OR 0.48, 95% CI: 0.31–0.76, *p* = 0.002; Table S3), confirming the nonlinear association.

**FIGURE 1:**
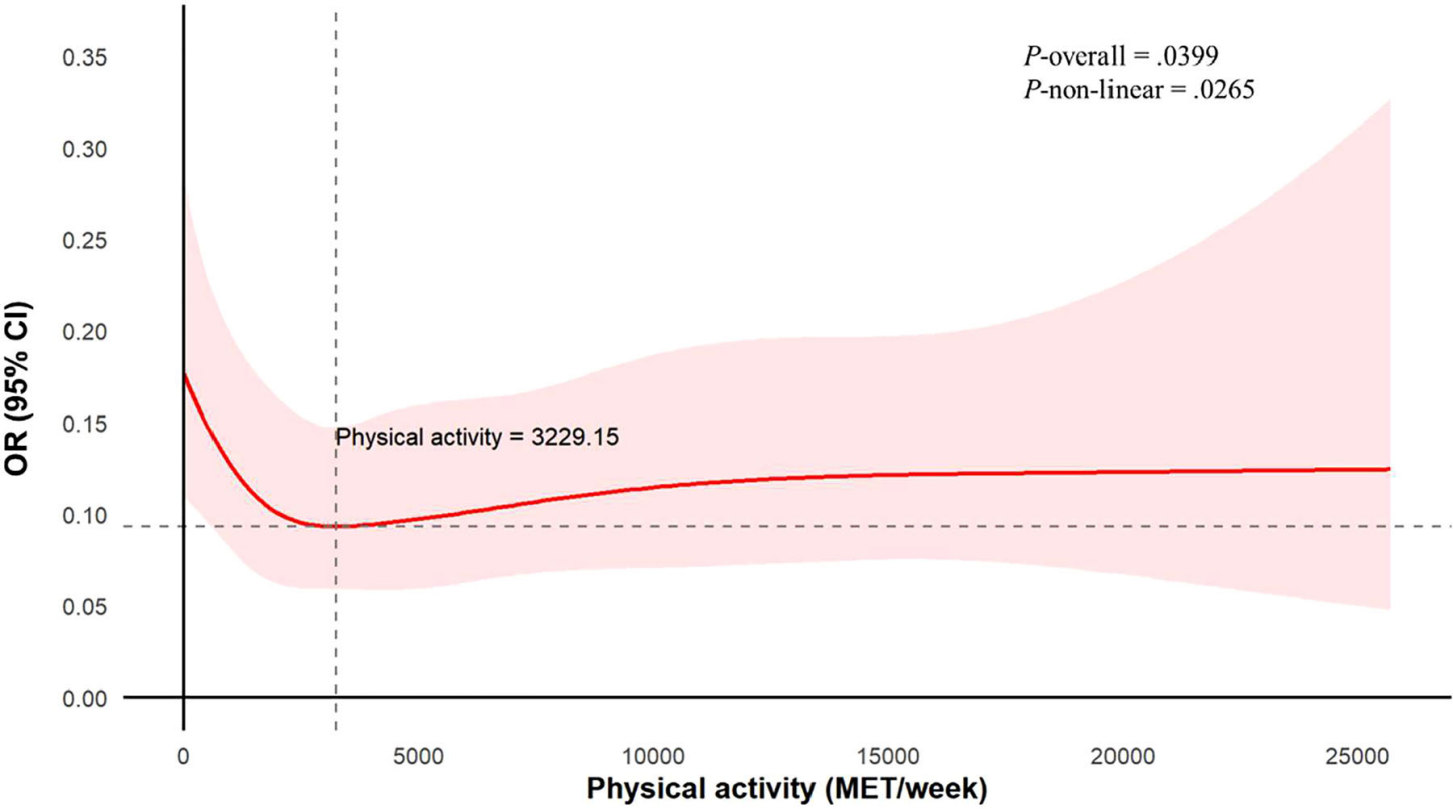
Dose‒response relationship between physical activity and the risk of cognitive impairment. Notes: OR = odds ratio; CI = confidence interval.

Furthermore, the RCS analysis suggested a nonlinear, L‐shaped association between physical activity and the risk of cognitive impairment in Wave 5 (*P*‐non‐linear = 0.0265), with an apparent turning point at approximately 3229 MET‐min/week (Figure 1). Below this level, higher physical activity was associated with a lower risk of cognitive impairment. Beyond this point, the curve appeared to plateau; however, the confidence intervals became wider at higher levels of physical activity, indicating greater statistical uncertainty. Therefore, the estimated threshold should be interpreted cautiously rather than as a definitive cut‐off for maximum benefit.

### 3.3 Identification and Descriptive Characteristics of Physical Activity Trajectories across Waves 1–5

**FIGURE 2:**
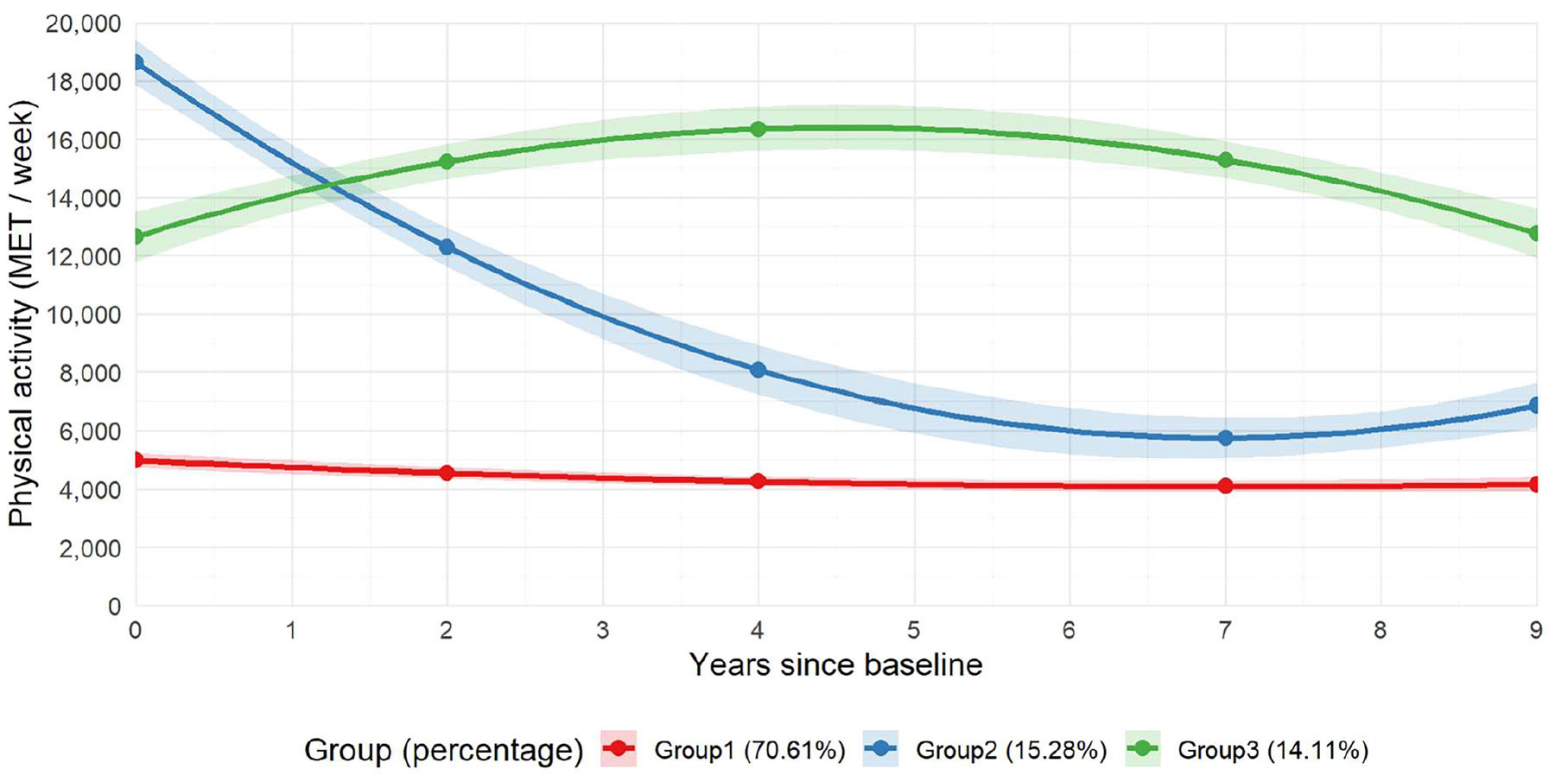
Physical activity trajectories identified by the GBTM model. Notes: Group 1 indicates the slight decline group; Group 2, the sharp drop group; and Group 3, the rise–then–fall group.

The GBTM revealed three groups over the course of 9 years (2011–2020): “slight‐decline (a relatively stable and modest linear decrease, *n* = 1451, 70.61%)”, “sharp‐drop (a rapid initial decline followed by a slower tapering, *n* = 314, 15.28%)”, and “rise‐then‐fall (a gradual increase and then a subsequent decline, an inverted U‐shaped curve, *n* = 290, 14.11%)” (Figure 2). These distinct trajectories capture the heterogeneity in physical activity behavior over time within this population.

Table 1 presents the sociodemographic and health‐related characteristics of participants in different physical activity trajectory groups in 2020. Post hoc analyses revealed that, compared with the slight decline group, both the sharp drop and rise–then–fall groups had higher proportions of men, younger age, rural residence, and current smoking. Baseline self‐reported hearing status (fair vs poor) did not differ significantly across the three trajectory groups (*p* = 0.884). At baseline, both the sharp drop and rise–then–fall groups had lower total cognitive function and mental state scores than the slight‐decline group (*p* = 0.003 and *p* < 0.001, respectively), whereas baseline episodic memory scores did not differ significantly across trajectory groups (*p* = 0.143). The rise–then–fall group had lower total cognitive function and mental state scores than the slight‐decline group (*p* = 0.005 and *p* < 0.001, respectively), whereas episodic memory scores did not differ significantly across trajectory groups (*p* = 0.291).

**TABLE 1:** Participant characteristics by trajectory group (2020).

Characteristics	All (%)	Slight decline group (*n* = 1451)	Sharp drop group (*n* = 314)	Rise‐then‐fall group (*n* = 290)	*P*
**Sex**					< 0.001
*Male*	959 (46.67)	619 (42.66) ^a^	174 (55.41) ^b^	166 (57.24) ^b^	
*Female*	1096 (53.33)	832 (57.34) ^a^	140 (44.59) ^b^	124 (42.76) ^b^	
**Age**					< 0.001
*54–59*	468 (22.77)	288 (19.85) ^a^	89 (28.34) ^b^	91 (31.38) ^c^	
*60–69*	852 (41.46)	585 (40.32) ^a^	123 (39.17) ^b^	144 (49.66) ^c^	
*70–101*	735 (35.77)	578 (39.83) ^a^	102 (32.48) ^b^	55 (18.97) ^c^	
**Place of residence**					< 0.001
*Village*	1457 (70.90)	935 (64.44) ^a^	269 (85.67) ^b^	253 (87.24) ^b^	
*City/town*	598 (29.10)	516 (35.56) ^a^	45 (14.33) ^b^	37 (12.76) ^b^	
**Education**					0.029
*Less than middle school*	1359 (66.13)	937 (64.58) ^a^	227 (72.29) ^b, c^	195 (67.24) ^a, c^	
*Middle school and above*	696 (33.87)	514 (35.42) ^a^	87 (27.71) ^b, c^	95 (32.76) ^a, c^	
**Marital status**					0.001
*Single*	490 (23.84)	377 (25.98) ^a^	64 (20.38) ^a, c^	49 (16.90) ^b, c^	
*Married*	1,565 (76.16)	1,074 (74.02) ^a^	250 (79.62) ^a, c^	241 (83.10) ^b, c^	
**Smoking status**					< 0.001
*Former*	328 (15.96)	210 (14.47) ^a^	62 (19.75) ^b^	56 (19.31) ^b^	
*Never*	1266 (61.61)	946 (65.20) ^a^	173 (55.10) ^b^	147 (50.69) ^b^	
*Now*	461 (22.43)	295 (20.33) ^a^	79 (25.16) ^b^	87 (30.00) ^b^	
**Drink status**					< 0.001
*No*	1399 (68.08)	1033 (71.19) ^a^	208 (66.24) ^a^	158 (54.48) ^b^	
*< Once per month*	192 (9.34)	131 (9.03) ^a^	25 (7.96) ^a^	36 (12.41) ^b^	
*≥ Once per month*	464 (22.58)	287 (19.78) ^a^	81 (25.80) ^a^	96 (33.10) ^b^	
**Sleep duration**					0.618
*< 7 h*	1315 (64.65)	933 (65.02)	204 (65.59)	178 (61.81)	
*7–9 h*	638 (31.37)	450 (31.36)	93 (29.90)	95 (32.99)	
*> 9 h*	81 (3.98)	52 (3.62)	14 (4.50)	15 (5.21)	
**Nap duration**					0.007
*0 min*	719 (35.19)	488 (33.87) ^a^	103 (32.91) ^a^	128 (44.29) ^b^	
*≤ 60 min*	907 (44.40)	649 (45.04) ^a^	140 (44.73) ^a^	118 (40.83) ^b^	
*> 60 min*	417 (20.41)	304 (21.10) ^a^	70 (22.36) ^a^	43 (14.88) ^b^	
**Chronic disease comorbidity**					0.390
*0*	1261 (61.36)	893 (61.54)	180 (57.32)	188 (64.83)	
*1–2*	717 (34.89)	503 (34.67)	120 (38.22)	94 (32.41)	
*≥ 3*	77 (3.75)	55 (3.79)	14 (4.46)	8 (2.76)	
**Depressive symptoms**					0.008
*No*	1312 (63.94)	957 (66.05) ^a^	186 (59.24) ^a, c^	169 (58.48) ^b, c^	
*Yes*	740 (36.06)	492 (33.95) ^a^	128 (40.76) ^a, c^	120 (41.52) ^b, c^	
**Hearing loss (baseline)**					0.884
*Fair*	1631 (79.37)	1154 (79.53)	250 (79.62)	227 (78.28)	
*Poor*	424 (20.63)	297 (20.47)	64 (20.38)	63 (21.72)	
**Total cognitive function score (baseline)**	12 (9.5, 14.5)	12.5 (10, 14.5) ^a^	12 (9, 14) ^b^	12 (9, 14) ^b^	0.003
**Episodic memory score (baseline)**	4 (3, 5)	4 (3, 5)	4 (3, 4.5)	4 (3, 4.5)	0.143
**Mental state score (baseline)**	9 (7, 10)	9 (7, 10) ^a^	9 (6, 10) ^b^	8 (6, 10) ^b^	< 0.001
**Total cognitive function score**	10.5 (7.5, 12.5)	10.5 (8, 12.5) ^a^	10.0 (7.5, 12) ^a, b^	9.5 (7.5, 12) ^b^	0.005
**Episodic memory score**	5.5 (4, 6.5)	5.5 (4, 7)	5.5 (3.5, 6.5)	5.5 (4, 6.5)	0.291
**Mental state score**	5 (4, 6)	5 (4, 6) ^a^	5 (4, 6) ^b^	5 (3, 5) ^b^	< 0.001

*Note*: Missing data were as follows: sleep duration, *n* = 21 (1.02%); nap duration, *n* = 12 (0.58%); and depressive symptoms, *n* = 3 (0.15%). Total cognitive function score, episodic memory score, and mental state score were continuous variables with skewed distributions, expressed as median (P25, P75). Values in the same row with different superscripts (a, b, c) differ significantly (Bonferroni corrected *P*< 0.05).

### 3.4 Associations Between Physical Activity Trajectories across Waves 1–5 and Cognitive Function in Wave 5

Logistic regression models showed no statistically significant associations between physical activity trajectory groups and cognitive impairment in Wave 5 (Table S5). In the pre‐specified analyses using continuous cognitive outcomes, multivariable linear regression models showed that, compared with the slight decline group, the rise‐then‐fall group presented significantly lower total cognitive scores (*β* = −0.621; 95% CI, −1.022–−0.219; *p* = 0.002) and mental state scores (*β* = −0.449; 95% CI, −0.648–−0.249; *p* < 0.001, Table 2). Although modest in absolute terms, these differences corresponded to approximately 3.0% of the total cognitive scale range (0‐21) and 4.1% of the mental status scale range (0‐11), respectively. The adjusted mean cognitive scores also supported this finding, particularly in the mental state domain (*p* < 0.001, Figure S2). In fully adjusted linear regression models examining the main and interactive effects of physical activity trajectory and high physical activity exposure (defined as activity ≥ the RCS‐derived threshold in at least three of Waves 1–4), the rise‐then‐fall trajectory remained significantly associated with lower cognitive function scores across all three domains: total cognition (*β* = −1.261; 95% CI, −2.113–−0.410; *p* = 0.004), episodic memory (*β* = −0.658; 95% CI, −1.258–−0.058; *p* = 0.032), and mental state (*β* = −0.603; 95% CI, −1.027–−0.179; *p* = 0.005; Table S6). However, the interaction terms between the trajectory and high physical activity exposure were not statistically significant (all *p* > 0.05), indicating that high activity exposure did not significantly modify the association between the rise‐then‐fall trajectory and cognitive function.

**TABLE 2:** Multivariate linear regression analysis of physical activity trajectories and cognitive function scores.

	Model 1		Model 2		Model 3	
	*β* (95% CI)	*P*	*β* (95% CI)	*P*	*β* (95% CI)	*P*
**Total cognitive function score**						
*Group 1*	Reference		Reference		Reference	
*Group 2*	−0.292 (−0.675–0.092)	0.136	−0.335 (−0.721–0.049)	0.087	−0.286 (−0.669–0.095)	0.141
*Group 3*	−0.632 (−1.033–−0.231)	0.002	−0.687 (−1.092–−0.282)	0.001	−0.621 (−1.022–−0.219)	0.002
**Episodic memory score**						
*Group 1*	Reference		Reference		Reference	
*Group 2*	−0.149 (−0.418–0.120)	0.278	−0.172 (–0.443–0.097)	0.211	−0.145 (−0.415–0.123)	0.288
*Group 3*	−0.174 (–0.455–0.108)	0.226	−0.211 (–0.495–0.073)	0.145	−0.171 (−0.455–0.111)	0.234
**Mental state score**						
*Group 1*	Reference		Reference		Reference	
*Group 2*	−0.142 (−0.333–0.048)	0.143	−0.162 (–0.354–0.028)	0.095	−0.141 (−0.331–0.049)	0.145
*Group 3*	−0.458 (−0.658–−0.259)	< 0.001	−0.476 (–0.677–−0.275)	< 0.001	−0.449 (−0.648–−0.249)	< 0.001

*Note*: Model 1: adjusted for sex, age, residence, education level, and marital status. Model 2: Smoking status, drinking status, sleep duration, and nap duration were added to Model 1. Model 3: Adding the number of chronic diseases and depressive symptoms to Model 2.

In addition, Table S7 shows that both the sharp drop and the rise‐then‐fall groups tend to be male, younger, and rural residents than the slight decline group (all *p* < 0.05). In addition, participants in the sharp drop group tend to be less educated; participants in the rise‐then‐fall group tend to be nonnapper, drinker, and exhibit depressive symptoms (all *P* <  0.05).

### 3.5 Sensitivity Analyses

**FIGURE 3:**
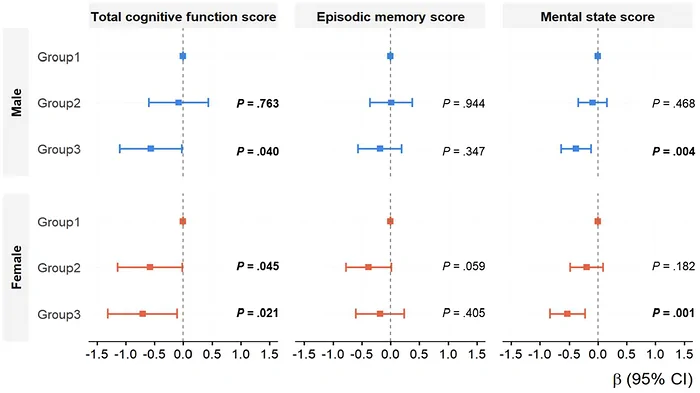
Sex‐stratified associations between physical activity trajectories and cognitive function domains. *Note*: Model adjusted for sex, age, place of residence, education level, marital status, smoking status, drinking status, sleep duration, nap duration, number of chronic diseases, and depressive symptoms. Group 1 represents the slight decline group; Group 2 represents the sharp drop group; and Group 3 represents the rise–then–fall group. CI = confidence interval.

Sensitivity analyses confirmed the robustness of the findings. Although significant differences in age, place of residence, marital status, sleep duration, chronic disease, and depressive symptoms were observed between participants included in the analytic sample and those excluded due to missing data or loss to follow‐up (all *p* < 0.05, Table S8), stratified analyses by sex, age, and education showed consistent trends across subgroups, with the rise–then–fall group remaining associated with lower total cognitive and mental state scores but not episodic memory (sex‐stratified results in Figure 3). Additional subgroup results, inverse probability of treatment weighting (IPTW)–adjusted estimates, and E‐value analyses are provided in the Supporting Information (Tables S9–S13; Figure S3). After additional adjustment for baseline cognitive function, the associations were attenuated but remained generally consistent in direction with the main analyses; the rise–then–fall group remained significantly associated with lower total cognitive function and mental state scores (Table S14).

## 4 Discussion

In this nationally representative 9‐year cohort of Chinese adults, we identified substantial heterogeneity in long‐term physical activity trajectories, including slight decline, sharp drop, and rise‐then‐fall. These trajectories describe distinct patterns of change in physical activity over time, rather than single‐time‐point activity levels, and therefore provide information on the stability, decline, or fluctuation of activity behaviours across follow‐up. Importantly, participants in the rise‐then‐fall trajectory had lower cognitive function in Wave 5, particularly mental status, than those in the slight‐decline trajectory. Because the trajectory groups represent descriptive patterns of physical activity over time, these associations should not be interpreted as causal effects of trajectory membership. Moreover, because Wave 5 physical activity partly contributed to trajectory classification, these findings should be interpreted as associations between long‐term physical activity patterns up to the time of cognitive assessment and cognitive status in Wave 5, rather than as fully prospective or predictive associations. In addition, our cross‐sectional results based on Wave 5 suggested a possible nonlinear association between physical activity and cognitive impairment among people with hearing loss.

The cross‐sectional RCS analysis in Wave 5 suggested a possible L‐shaped association between physical activity and cognitive impairment, which is broadly consistent with previous systematic reviews and cohort studies reporting nonlinear associations between physical activity and cognitive outcomes (Gallardo‐Gómez et al. 2022; Bull et al. 2020; You et al. 2023; Dong et al. 2025). Before the apparent turning point of approximately 3229 MET‐min/week, higher physical activity was associated with a lower risk of cognitive impairment; beyond this level, the curve appeared to plateau. However, because the confidence intervals widened at higher levels of physical activity, this apparent plateau and the estimated threshold should be interpreted cautiously and regarded as exploratory rather than as a definitive cut‐off for maximum benefit. The threshold observed in our study was slightly higher than the commonly reported range of 600–3000 MET‐min/week, which may be partly related to differences in population characteristics. Adults with hearing loss may require greater attentional and executive resources during physical activity and may experience greater cognitive–motor interference and higher dual‐task costs during movement (Wunderlich et al. 2024). In addition, hearing loss‐associated communication barriers and social withdrawal may lead to shorter, more fragmented physical activity patterns and a narrower range of activity types or settings (Assi et al. 2024), which may partly explain why a higher cumulative activity dose was observed in this population. Overall, this study provides exploratory evidence of an apparent physical activity turning point observed within adults with self‐reported hearing loss, extending current knowledge by focusing on within‐population heterogeneity rather than between‐group differences by hearing status.

Our findings support the fact that physical activity decreases gradually during aging, as the slight decline trajectory accounts for the majority of participants. These findings are broadly consistent with a previous CHARLS study of the general population aged 45 years and older, in which sharp‐decline and unstable increasing trajectories accounted for 13.7% and 12.1% of participants, respectively (Wang et al. 2024). In the present study of adults with self‐reported hearing loss, the corresponding proportions were 15.28% and 14.11%, suggesting that unstable physical activity patterns may also be evident in this subgroup. However, because this comparison was based on published findings rather than a hearing‐normal comparison group analysed under the same framework, we cannot conclude that hearing loss amplifies age‐related fluctuations in activity patterns. These hypothesis‐generating interpretations are intended to provide possible explanations for the rise–then–fall trajectory, rather than conclusions directly supported by our data. The earlier increase in physical activity may have partly reflected broader contextual influences, such as China's National Fitness Plans (2011–2015 and 2016–2020), and increased awareness of the impact of hearing loss on daily life (McCambridge et al. 2014; Zheng et al. 2022). The subsequent decrease in physical activity may be related to ageing‐related health changes or adverse health events, given that altered physical activity is closely associated with ageing and specific diseases (Lounassalo et al. 2019), and that hearing loss has been associated with falls and cardiovascular diseases (Yeo et al. 2025; Huang et al. 2025). In addition, it is possible that compensatory behaviours driven by self‐awareness or policy incentives may not have been sustained in the context of increasing social withdrawal and psychological distress associated with ageing and hearing loss (Prieur Chaintré et al. 2024; Huang et al. 2024).

Given the association between the rise–then–fall trajectory and lower cognitive scores, we further performed exploratory analyses to identify the characteristics of this trajectory compared with the slight decline trajectory, including factors associated with both increases and decreases in physical activity (male sex, moderate drinking, regular napping, and good mental health for increased physical activity; female sex, older age, rural residence, and depressive symptoms for decreased physical activity) (Nemoto et al. 2024; Buvarp et al. 2023; Gudmundsson et al. 2015; Finkel et al. 2018; Henderson et al. 2023; Kong et al. 2022). These characteristics may provide clues for understanding this unstable trajectory, but the underlying behavioural and psychological mechanisms were not directly evaluated in this study. In the “rise‐then‐fall” trajectory, the earlier increase may reflect an unsustainable compensatory activity pattern among some participants. For example, younger rural men may have higher physical activity levels due to work and social roles. At the same time, depressive symptoms, poor sleep‐related behaviours, which have been linked to depression and chronic conditions such as obesity, diabetes, and cardiovascular disease (Ramos et al. 2023), and alcohol consumption may contribute to greater instability in activity engagement. However, these interpretations remain speculative and should be considered as possible contextual mechanisms rather than empirical findings derived from the present analyses. Therefore, improving community facilities and enhancing social support and mental health services may help create environments conducive to sustained physical activity, which could be relevant to cognitive health among adults with self‐reported hearing loss.

The nonsignificant association between the sharp‐drop trajectory and cognitive outcomes may be partly explained by the possibility that participants with exceptionally high baseline activity had accumulated cardiovascular and neural advantages, as sustained high activity improves endothelial function and cerebral vascular regulation (Szczegielniak and Krivošová 2025). This finding is consistent with the possibility that accumulated physiological reserves may buffer later cognitive vulnerability, thereby contributing to cognitive resilience (Canton‐Martínez et al. 2022; Kerr et al. 2024; Zhang et al. 2024). Furthermore, the inconsistent findings between logistic regression using cognitive impairment as a binary outcome and linear regression using continuous cognitive scores may reflect information loss and reduced statistical power after dichotomizing cognition. Continuous cognitive scores preserve variation across the full distribution and may therefore be more sensitive to subtle cognitive differences. Thus, the significant associations observed for continuous cognitive scores should be interpreted as modest differences in cognitive performance rather than clear differences in clinically defined cognitive impairment. This is particularly relevant for individuals with hearing loss, who may experience heightened auditory processing demands and earlier cognitive vulnerability (Luijendijk et al. 2023; Wayne and Johnsrude 2015; Golub et al. 2020; Yu et al. 2024). Multiple sensitivity analyses also ensure the robustness of our results.

Our study has several limitations. First, hearing loss was assessed as a binary self‐reported variable because of the low prevalence of hearing aid use and limited information on hearing loss severity, which prevented further analysis of hearing loss severity and hearing aid use. In addition, because some cognitive tasks were verbally administered and CHARLS did not provide detailed information on accommodations for participants with hearing loss, hearing impairment itself may have affected cognitive test performance and introduced measurement bias. Second, because this study focused on heterogeneity in long‐term physical activity patterns among adults with self‐reported hearing loss, the analysis was intentionally restricted to this subgroup and did not include a non‐hearing‐impaired comparison cohort. Therefore, the findings should be interpreted as within‐group evidence and cannot establish whether the observed associations are unique to adults with hearing loss. Third, although participants with baseline cognitive impairment were excluded and sensitivity analyses additionally adjusted for baseline cognitive function, residual confounding by pre‐existing cognitive differences cannot be fully excluded given the temporal stability of cognitive performance. In addition, declining cognitive function during follow‐up may have influenced subsequent physical activity patterns, particularly in trajectory analyses; therefore, reverse causality cannot be fully excluded. Moreover, because Wave 5 physical activity contributed to trajectory classification and was measured contemporaneously with cognitive function, the trajectory analysis was not fully prospective, which limits the temporal interpretation of the observed associations. Fourth, physical activity duration was assessed using categorical responses and converted into approximate continuous values for MET‐min/week calculation. This approach may have introduced measurement error, particularly because the open‐ended highest category (> 4 h) was assigned 4 h, potentially underestimating activity volume among highly active participants. Fifth, although IPTW and multivariable adjustments were applied, residual confounding cannot be fully excluded. Moreover, because several Wave 5 covariates, such as depressive symptoms, chronic disease burden, sleep duration, daytime napping, and alcohol use, may have been influenced by prior physical activity patterns, adjustment for these variables may have introduced overadjustment and attenuated the observed associations. In particular, the exclusion of participants who died or were lost to follow‐up may have affected the representativeness of the analytic sample, as hearing loss, physical activity, cognitive function, and mortality are interrelated.

## 5 Conclusions

We observed an L‐shaped association between physical activity and cognitive impairment in people with hearing loss and further demonstrated heterogeneity in long‐term physical activity trajectories. Specifically, participants with the “rise–then–fall” pattern had lower cognitive function and were more likely to be male, younger, rural‐dwelling, frequent drinkers, non‐nappers, and to experience depressive symptoms. Maintaining stable long‐term physical activity may be relevant to cognitive health in individuals with hearing loss. Therefore, creating environments favourable for sustained physical activity, such as enhancing community facilities, strengthening social support and mental health services, and implementing policies that encourage regular physical activity, may support cognitive health in this population, thereby contributing to healthy ageing. As this study was intentionally restricted to adults with self‐reported hearing loss, these findings should be interpreted as within‐group evidence rather than as evidence that the observed associations are unique to adults with hearing loss.

## Author Contributions

**Xiaoyang Li**: conceptualization, methodology, data curation, formal analysis, Writing – original draft. **Ruxia Qiu**: writing – review and editing. **Yingxin Tang**: writing – review and editing. **Chi Zhang**: writing – review and editing. **Yinan Zhao**: writing – review and editing, supervision. **Hui Feng**: supervision, writing – review and editing. **Xiaoling Zhang**: writing – review and editing, funding acquisition.

## Funding

This work was funded by the Major Scientific Research Special Project of National Key Clinical Specialties of Hunan Provincial Health Commission (2023 Annual) (Z2023073, Xiaoling Zhang), the National Natural Science Foundation of China (72374224, Hui Feng), and Central South University Advanced Interdisciplinary Research Programme (2023QYJC034, Hui Feng).

## Ethics Statement

This study is a secondary analysis of publicly available datasets and was therefore exempt from additional Institutional Review Board review. The original CHARLS study obtained ethical approval from the Ethical Review Committee of Peking University (IRB00001052‐11015). All procedures were conducted in accordance with the ethical principles outlined in the Declaration of Helsinki (2013 revision) and relevant institutional guidelines and regulations.

## Consent

All participants were informed about the purpose of the survey, assured of confidentiality, and provided written informed consent prior to participation. Participation was voluntary and could be withdrawn at any time without consequence.

## Conflicts of Interest

The authors declare no conflicts of interest.

## Supporting information




**Supplementary Materials**: brb371646‐sup‐0001‐SuppMat.docx

## Data Availability

The data used in this study are publicly available from the China Health and Retirement Longitudinal Study (CHARLS) at http://charls.pku.edu.cn/. Therefore, no additional data are available from the authors.
