# Supplementary material for: Physical Activity and Cognitive Function Among Chinese Adults Aged 45 Years and Older with Self‐Reported Hearing Loss: A 9‐Year Longitudinal Study
Source: Brain Behav. 2026 Aug 20;16(8):e71646. doi: 10.1002/brb3.71646 (PMC13493363; doi:10.1002/brb3.71646)
Supplement: Supplementary file 1 [file BRB3-16-e71646-s001.docx]

**Supplementary Materials**

**Supplementary Methods and Results: Model Selection for Physical Activity Trajectories**

To determine the optimal number of physical activity trajectories, we compared models with two to five latent classes. Selection followed established criteria (1): lower Bayesian Information Criterion (BIC) values indicated better fit; higher-order polynomial terms were retained only when statistically significant; average posterior probabilities of assignment (AvePP) >0.70 signaled adequate classification; and each group was required to include at least 5% of the sample to ensure stability. Based on these indices, the three-class model provided the best balance between statistical fit and interpretability. As shown in Table S4, the three-group model yielded the lowest BIC (176085.45) and AvePP values >0.70 for all groups, supporting its selection as the optimal solution.

**Sensitivity analyses**

Methods: Subgroup consistency was evaluated by conducting stratified analyses by sex, age and education level to assess subgroup consistency. Furthermore, an inverse-probability-of-treatment weighting (IPTW) sensitivity analysis was performed: 1) Multinomial logistic regression was used to estimate the probability distributions of the three physical activity trajectories at the individual level, and thereby to construct IPTW weights; 2) The weights were truncated at the 1st and 99th percentiles to mitigate the influence of extreme values; 3) we used these truncated weights to rerun our multivariable linear regression to compare effect estimates before and after weighting, thereby confirming the robustness of our findings. Finally, E-value was calculated to quantify the potential impact of unmeasured confounding variables (2). In addition, to examine whether the observed trajectory–cognition associations were influenced by pre-existing cognitive differences, the main trajectory–cognition models were further adjusted for baseline cognitive function.

Results: Subgroup analysis results are presented in the main text. The IPTW-adjusted models produced results consistent with the main analysis, with moderately attenuated estimates (Figure S3, Table S12). E-values were moderate to large for the rise–then–fall group (e.g. 1.638 for total cognition and 1.871 for mental state), indicating that substantial unmeasured confounding would be required to explain away the observed associations, whereas estimates for the sharp drop group were smaller (Table S13). After additional adjustment for baseline cognitive function, the associations were attenuated but remained generally consistent in direction with the main analyses. In particular, the rise–then–fall group remained significantly associated with lower total cognitive function and mental state scores, whereas the associations with cognitive impairment and episodic memory remained nonsignificant (Table S14).

**List of Figures**

Figure S1. Flow diagram of the study population

Figure S2. Adjusted predicted cognitive scores by physical activity trajectory group

Figure S3. Comparison of original and IPTW-weighted *β* (95% CI) estimates across cognitive domains

**List of Tables**

Table S1. STROBE Statement—Checklist of items that should be included in reports of cohort studies

Table S2. Sociodemographic characteristics of participants (2020)

Table S3. Association between physical activity quintiles and cognitive impairment

Table S4. Model parameters for trajectory fitting of physical activity over time

Table S5. Multivariable logistic regression of physical activity trajectories and cognitive impairment

Table S6. Main and interaction effects of physical activity trajectory and high physical activity exposure on cognitive function

Table S7. Multivariate logistic regression results of factors associated with physical activity trajectories.

Table S8. Baseline sociodemographic characteristics of participants included and not included in the analytic sample

Table S9. Sex‐stratified associations between physical activity trajectories and cognitive function

Table S10. Age‐stratified associations between physical activity trajectories and cognitive function

Table S11. Education‐stratified associations between physical activity trajectories and cognitive function

Table S12. Multivariable linear regression of physical activity trajectories on cognitive scores using truncated IPTW (1st–99th percentiles)

Table S13. E values for the association between physical activity trajectories on cognitive scores

Table S14. Sensitivity analysis of the associations between physical activity trajectories and cognitive outcomes after additional adjustment for baseline cognitive function

Figure S1. Flow diagram of the study population

Figure S2. Adjusted predicted cognitive scores by physical activity trajectory group

Notes: Values are predicted means (95% CI) from fully adjusted linear regression models. Group 1 indicates the slight decline group; Group 2, the sharp drop group; and Group 3, the rise–then–fall group. ** *P* < .01; *** *P* < .001.

Figure S3. Comparison of original and IPTW-weighted *β* (95% CI) estimates across cognitive domains

Note: Model adjusted for sex, age, place of residence, education level, marital status, smoking status, drinking status, sleep duration, nap duration, the number of chronic diseases and depressive symptoms. Group 1 indicates the slight decline group; Group 2, the sharp drop group; and Group 3, the rise–then–fall group. CI = Confidence Interval.

Table S1. STROBE Statement—Checklist of items that should be included in reports of cohort studies

|  | **Item No** | **Recommendation** | **Location in Manuscript** |
| --- | --- | --- | --- |
| **Title and abstract** | 1 | (a) Indicate the study’s design with a commonly used term in the title or the abstract | Page 4, 49 |
|  |  | (b) Provide in the abstract an informative and balanced summary of what was done and what was found | Pages 4-5, 62-74 |
| **Introduction** | | |  |
| Background/rationale | 2 | Explain the scientific background and rationale for the investigation being reported | Introduction, paragraphs 1-4 |
| Objectives | 3 | State specific objectives, including any prespecified hypotheses | Introduction, final paragraph |
| **Methods** | | |  |
| Study design | 4 | Present key elements of study design early in the paper | Methods, Study Participants |
| Setting | 5 | Describe the setting, locations, and relevant dates, including periods of recruitment, exposure, follow-up, and data collection | Methods, Study Participants |
| Participants | 6 | (a) Give the eligibility criteria, and the sources and methods of selection of participants. Describe methods of follow-up | Methods, Study Participants |
|  |  | (b) For matched studies, give matching criteria and number of exposed and unexposed | Methods, Study Participants |
| Variables | 7 | Clearly define all outcomes, exposures, predictors, potential confounders, and effect modifiers. Give diagnostic criteria, if applicable | Methods, Physical Activity Measurement, Cognitive Function Measurement, Covariates |
| Data sources/ measurement | 8* | For each variable of interest, give sources of data and details of methods of assessment (measurement). Describe comparability of assessment methods if there is more than one group | Methods, Physical Activity Measurement, Cognitive Function Measurement, Covariates |
| Bias | 9 | Describe any efforts to address potential sources of bias | Methods, Statistical Analysis |
| Study size | 10 | Explain how the study size was arrived at | Methods, Study Participants |
| Quantitative variables | 11 | Explain how quantitative variables were handled in the analyses. If applicable, describe which groupings were chosen and why | Methods, Statistical Analysis |
| Statistical methods | 12 | (a) Describe all statistical methods, including those used to control for confounding | Methods, Statistical Analysis |
|  |  | (b) Describe any methods used to examine subgroups and interactions | Methods, Statistical Analysis |
|  |  | (c) Explain how missing data were addressed | Methods, Study Participants |
|  |  | (d) If applicable, explain how loss to follow-up was addressed | Methods, Study Participants |
|  |  | (e) Describe any sensitivity analyses |  |
| **Results** | | |  |
| Participants | 13* | (a) Report numbers of individuals at each stage of study—eg numbers potentially eligible, examined for eligibility, confirmed eligible, included in the study, completing follow-up, and analysed | Methods, Study Participants  Results, Demographic characteristics of participants |
|  |  | (b) Give reasons for non-participation at each stage | Methods, Study Participants  Results |
|  |  | (c) Consider use of a flow diagram | Figure S1 in the Supplement |
| Descriptive data | 14* | (a) Give characteristics of study participants (eg demographic, clinical, social) and information on exposures and potential confounders | Table S2 in the Supplement |
|  |  | (b) Indicate number of participants with missing data for each variable of interest | Table 1, Table S2, Table S8 |
|  |  | (c) Summarise follow-up time (eg, average and total amount) | Not applicable; follow-up duration defined by CHARLS waves (2011–2020). |
| Outcome data | 15* | Report numbers of outcome events or summary measures over time | Not applicable; cognitive function was analyzed as a continuous outcome measured at the final follow-up (2020) rather than as repeated events over time. |
| Main results | 16 | (a) Give unadjusted estimates and, if applicable, confounder-adjusted estimates and their precision (eg, 95% confidence interval). Make clear which confounders were adjusted for and why they were included | Table 2, Table S3, Table S5, Table S6, Table S9, Table S10, Table S11, Table S12 |
|  |  | (b) Report category boundaries when continuous variables were categorized | Category boundaries for continuous variables (e.g., physical activity quintiles Q1–Q5) were reported in the Methods |
|  |  | (c) If relevant, consider translating estimates of relative risk into absolute risk for a meaningful time period | Not applicable |
| Other analyses | 17 | Report other analyses done—eg analyses of subgroups and interactions, and sensitivity analyses | Sensitivity analyses conducted. |
| **Discussion** | | |  |
| Key results | 18 | Summarise key results with reference to study objectives | Main results (L-shaped association; 3 trajectories; rise–then–fall linked to decline) summarised in Discussion |
| Limitations | 19 | Discuss limitations of the study, taking into account sources of potential bias or imprecision. Discuss both direction and magnitude of any potential bias | Study limitations were discussed |
| Interpretation | 20 | Give a cautious overall interpretation of results considering objectives, limitations, multiplicity of analyses, results from similar studies, and other relevant evidence | Overall interpretation aligned with aims and prior evidence, acknowledging limitations. |
| Generalisability | 21 | Discuss the generalisability (external validity) of the study results | Findings are based on nationally representative CHARLS data, supporting generalisability to middle-aged and older Chinese adults, though external validity to other populations may be limited. |
| **Other information** | | |  |
| Funding | 22 | Give the source of funding and the role of the funders for the present study and, if applicable, for the original study on which the present article is based | This work was funded by the Major Scientific Research Special Project of National Key Clinical Specialties of Hunan Provincial Health Commission (2023 Annual) (Z2023073, Xiaoling Zhang), the National Natural Science Foundation of China (72374224, Hui Feng), and Central South University Advanced Interdisciplinary Research Programme (2023QYJC034, Hui Feng). |

*Give information separately for exposed and unexposed groups.

Table S2. Sociodemographic characteristics of participants (2020)

| Characteristics | All (%) | Cognitive normal  (*n* = 1,774) | Cognitive impairment  (*n* = 281) | *P* |
| --- | --- | --- | --- | --- |
| **Sex** |  |  |  | .118 |
| *Male* | 959 (46.67) | 840 (47.35) | 119 (42.35) |  |
| *Female* | 1,096 (53.33) | 934 (52.65) | 162 (57.65) |  |
| **Age** |  |  |  | < .001 |
| *54-59* | 468 (22.77) | 441 (24.86) | 27 (9.61) |  |
| *60-69* | 852 (41.46) | 764 (43.09) | 88 (31.32) |  |
| *70-101* | 735 (35.77) | 569 (32.09) | 166 (59.07) |  |
| **Place of residence** |  |  |  | < .001 |
| *Village* | 1,457 (70.90) | 1,228 (69.22) | 229 (81.49) |  |
| *City/Town* | 598 (29.10) | 546 (30.78) | 52 (18.51) |  |
| **Education** |  |  |  | < .001 |
| *Less than middle school* | 1,359 (66.13) | 1,098 (61.89) | 261 (92.88) |  |
| *Middle school and above* | 696 (33.87) | 676 (38.11) | 20 (7.12) |  |
| **Marital status** |  |  |  | < .001 |
| *Single* | 490 (23.84) | 391 (22.04) | 99 (35.23) |  |
| *Married* | 1,565 (76.16) | 1,383 (77.96) | 182 (64.77) |  |
| **Smoking status** |  |  |  | .969 |
| *Former* | 328 (15.96) | 284 (16.01) | 44 (15.66) |  |
| *Never* | 1,266 (61.61) | 1,091 (61.50) | 175 (62.28) |  |
| *Now* | 461 (22.43) | 399 (22.49) | 62 (22.06) |  |
| **Drink status** |  |  |  | .003 |
| *No* | 1,399 (68.08) | 1,184 (66.74) | 215 (76.51) |  |
| *<once per month* | 192 (9.34) | 176 (9.92) | 16 (5.69) |  |
| *≥once per month* | 464 (22.58) | 414 (23.34) | 50 (17.79) |  |
| **Sleep duration** |  |  |  | .034 |
| *<7 h* | 1,315 (64.65) | 1,150 (65.38) | 165 (60.00) |  |
| *7-9 h* | 638 (31.37) | 546 (31.04) | 92 (33.45) |  |
| *>9 h* | 81 (3.98) | 63 (3.58) | 18 (6.55) |  |
| **Nap duration** |  |  |  | < .001 |
| *0 min* | 719 (35.19) | 624 (35.33) | 95 (34.30) |  |
| *≤60 min* | 907 (44.40) | 805 (45.58) | 102 (36.82) |  |
| *>60 min* | 417 (20.41) | 337 (19.08) | 80 (28.88) |  |
| **Chronic disease comorbidity** |  |  |  | .266 |
| *0* | 1,261 (61.36) | 1,087 (61.27) | 174 (61.92) |  |
| *1-2* | 717 (34.89) | 625 (35.23) | 92 (32.74) |  |
| *≥3* | 77 (3.75) | 62 (3.49) | 15 (5.34) |  |
| **Depressive symptoms** |  |  |  | < .001 |
| *No* | 1,312 (63.94) | 1,161 (65.45) | 151 (54.32) |  |
| *Yes* | 740 (36.06) | 613 (34.55) | 127 (45.68) |  |
| **Hearing loss (baseline)** |  |  |  | < .001 |
| *Fair* | 1,631 (79.37%) | 1,428 (80.50%) | 203 (72.24%) |  |
| *Poor* | 424 (20.63%) | 346 (19.50%) | 78 (27.76%) |  |
| **Total cognitive function score (baseline)** | 12 (9.5, 14.5) | 12.5 (10, 14.5) | 9.5 (7.5, 12) | < .001 |
| **Episodic memory score (baseline)** | 4 (3, 5) | 4 (3, 5) | 3 (2.5, 4.5) | < .001 |
| **Mental state score (baseline)** | 9 (7, 10) | 9 (7, 10) | 7 (5, 9) | < .001 |

Note: Missing data were as follows: sleep duration, *n* = 21 (1.02%); nap duration, *n* = 12 (0.58%); and depressive symptoms, *n* = 3 (0.15%).

Table S3. Association between physical activity quintiles and cognitive impairment

| Physical activity | *n* | Event (%) | OR for cognitive impairment (95% CI) | | |
| --- | --- | --- | --- | --- | --- |
|  |  |  | Model 1 | Model 2 | Model 3 |
| **Q1** | 418 | 82 (19.62) | (Reference) | (Reference) | (Reference) |
| **Q2** | 408 | 57 (13.97) | 0.79 (0.53 to 1.16) | 0.79 (0.53 to 1.18) | 0.81 (0.54 to 1.20) |
| **Q3** | 407 | 38 (9.34) | 0.53 (0.34 to 0.81) ** | 0.51 (0.32 to 0.79) ** | 0.48 (0.31 to 0.76) ** |
| **Q4** | 411 | 55 (13.38) | 0.76 (0.51 to 1.13) | 0.78 (0.52 to 1.16) | 0.78 (0.52 to 1.16) |
| **Q5** | 411 | 49 (11.92) | 0.70 (0.46 to 1.04) | 0.74 (0.48 to 1.12) | 0.73 (0.48 to 1.11) |

Note: Model 1: adjusted for sex, age, residence, education level, and marital status. Model 2: added smoking status, drinking status, sleep duration, and nap duration to model 1. Model 3: added number of chronic diseases and depressive symptoms to model 2. *******P* < .01, Q1 - Q5 represent quintiles of physical activity (MET-min/week), ranging from the lowest (Q1) to the highest (Q5) levels.

Table S4. Model parameters for trajectory fitting of physical activity over time

| Number of clusters | Each group order | Average posterior probability (AvePP) of each trajectory group | | | | | The proportion of people in each trajectory group (%) | | | | | BIC | AIC | LL |
| --- | --- | --- | --- | --- | --- | --- | --- | --- | --- | --- | --- | --- | --- | --- |
|  |  | Group 1 | Group 2 | Group 3 | Group 4 | Group 5 | Group 1 | Group 2 | Group 3 | Group 4 | Group 5 |  |  |  |
| 1 | 3 | 1 |  |  |  |  | 100 |  |  |  |  | 176846.72 | 176796.07 | –88389.04 |
| 1 | 1 | 1 |  |  |  |  | 100 |  |  |  |  | 176819.8 | 176797.28 | –88394.64 |
| 2 | 1, 1 | 0.8884 | 0.9483 |  |  |  | 24.77 | 75.23 |  |  |  | 176438.65 | 176399.25 | –88192.63 |
| 2 | 2, 2 | 0.8850 | 0.9496 |  |  |  | 24.96 | 75.04 |  |  |  | 176450.71 | 176400.06 | –88191.03 |
| 3 | 1, 1, 1 | 0.8168 | 0.9475 | 0.8316 |  |  | 16.11 | 71.48 | 12.41 |  |  | 176151.02 | 176094.74 | –88037.37 |
| 3 | 2, 2, 2 | 0.9462 | 0.8227 | 0.8618 |  |  | 70.32 | 15.72 | 13.97 |  |  | 176084.24 | 176011.07 | –87992.54 |
| **3** | **1, 2, 2** | **0.9459** | **0.8236** | **0.8579** |  |  | **70.61** | **15.28** | **14.11** |  |  | **176085.45** | **176017.92** | **–87996.96** |
| 4 | 1, 1, 1, 1 | 0.9475 | 0 | 0.8316 | 0.8168 |  | 71.48 | 0 | 12.41 | 16.11 |  | 176173.91 | 176100.74 | –88037.37 |
| 5 | 1, 1, 1, 1, 1 | 0.2061 | 0.2050 | 0.2056 | 0.2060 | 0.2049 | 14.65 | 26.33 | 6.67 | 20.24 | 32.12 | 176911.51 | 176821.46 | –88394.73 |

Note: AIC = Akaike Information Criterion; BIC = Bayesian Information Criterion; LL = Log-likelihood. The best-fitting model is indicated in bold.

Table S5. Multivariable logistic regression of physical activity trajectories and cognitive impairment

|  | Model 1 | | Model 2 | | Model 3 | |
| --- | --- | --- | --- | --- | --- | --- |
|  | OR (95% CI) | *P* | OR (95% CI) | *p* | OR (95% CI) | *P* |
| Group 1 | Reference | | Reference | | Reference | |
| Group 2 | 1.32  (0.92 to 1.89) | .130 | 1.39  (0.97 to 2.01) | .073 | 1.36  (0.94 to 1.96) | .098 |
| Group 3 | 1.30  (0.87 to 1.94) | .192 | 1.41  (0.94 to 2.12) | .097 | 1.35  (0.90 to 2.04) | .145 |

Note: Model 1 adjusted for sex, age, residence, education level, and marital status. Model 2 additionally adjusted for smoking status, drinking status, sleep duration, and nap duration. Model 3 further adjusted for number of chronic diseases and depressive symptoms. Group 1 indicates the slight decline trajectory; Group 2, the sharp drop trajectory; and Group 3, the rise–then–fall trajectory.

Table S6. Main and interaction effects of physical activity trajectory and high physical activity exposure on cognitive function

|  | Total cognitive function score | | Episodic memory score | | Mental state score | |
| --- | --- | --- | --- | --- | --- | --- |
|  | *β* (95% CI) | *P* | *β* (95% CI) | *P* | *β* (95% CI) | *P* |
| **Physical activity trajectory** | | | | | | |
| *Group 1* | Reference |  | Reference |  | Reference |  |
| *Group 2* | –0.532 (–1.132 to 0.067) | .082 | –0.260 (–0.682 to 0.162) | .227 | –0.272 (–0.570 to 0.026) | .074 |
| *Group 3* | –1.261 (–2.113 to –0.410) | **.004** | –0.658 (–1.258 to –0.058) | **.032** | –0.603 (–1.027 to –0.179) | .005 |
| **High PA exposure** | 0.171 (–0.202 to 0.544) | .369 | 0.246 (–0.016 to 0.510) | .066 | –0.075 (–0.261 to 0.110) | .424 |
| **Physical activity trajectory × High physical activity exposure** | | | | | | |
| *Group 1 ×High physical activity exposure* | Reference |  | Reference |  | Reference |  |
| *Group 2 ×High physical activity exposure* | 0.278 (–0.518 to 1.074) | .493 | 0.028 (–0.532 to 0.589) | .920 | 0.249 (–0.146 to 0.645) | .217 |
| *Group 3 ×High physical activity exposure* | 0.667 (–0.315 to 1.651) | .183 | 0.426 (–0.266 to 1.119) | .227 | 0.240 (–0.248 to 0.730) | .335 |

Note: Model adjusted for sex, age, place of residence, education level, marital status, smoking status, drinking status, sleep duration, nap duration, the number of chronic diseases and depressive symptoms. Group 1 indicates the slight decline trajectory; Group 2, the sharp drop trajectory; and Group 3, the rise–then–fall trajectory.

Table S7. Multivariate logistic regression results of factors associated with physical activity trajectories.

| Characteristics | Group 2 **vs.** Group 1 | | Group 3 **vs.** Group 1 | |
| --- | --- | --- | --- | --- |
|  | OR (95% CI) | *P* | OR (95% CI) | *P* |
| **Sex** |  |  |  |  |
| *Male* | Reference |  | Reference |  |
| *Female* | 0.50 (0.34 to 0.73) | < .001 | 0.61 (0.40 to 0.94) | .025 |
| **Age** |  |  |  |  |
| *54-59* |  |  |  |  |
| *60-69* | 0.52 (0.37 to 0.72) | < .001 | 0.62 (0.45 to 0.86) | .005 |
| *70-101* | 0.37 (0.26 to 0.53) | < .001 | 0.23 (0.15 to 0.34) | < .001 |
| **Place of residence** |  |  |  |  |
| *Village* | Reference |  | Reference |  |
| *City/Town* | 0.32 (0.23 to 0.46) | < .001 | 0.27 (0.18 to 0.39) | < .001 |
| **Education** |  |  |  |  |
| *Less than middle school* | Reference |  | Reference |  |
| *Middle school and above* | 0.59 (0.44 to 0.80) | .001 | 0.74 (0.55 to 1.00) | .055 |
| **Marital status** |  |  |  |  |
| *Single* | Reference |  | Reference |  |
| *Married* | 1.19 (0.87 to 1.65) | .272 | 1.21 (0.84 to 1.73) | .291 |
| **Smoking status** |  |  |  |  |
| *Former* | Reference |  | Reference |  |
| *Never* | 0.86 (0.57 to 1.30) | .488 | 0.74 (.46 to 1.17) | .198 |
| *Now* | 0.80 (0.54 to 1.19) | .284 | 0.85 (0.57 to 1.29) | .457 |
| **Drink status** |  |  |  |  |
| *No* | Reference |  | Reference |  |
| *<once per month* | 0.85 (0.53 to 1.37) | .526 | 1.78 (1.14 to 2.80) | .011 |
| *≥once per month* | 1.18 (0.84 to 1.66) | .327 | 1.87 (1.31 to 2.66) | .001 |
| **Sleep duration** |  |  |  |  |
| *<7 h* | Reference |  | Reference |  |
| *7-9 h* | 0.89 (0.67 to 1.19) | .450 | 1.08 (0.80 to 1.45) | .606 |
| *>9 h* | 0.98 (0.49 to 1.94) | .955 | 1.51 (0.74 to 3.09) | .254 |
| **Nap duration** |  |  |  |  |
| *0 min* | Reference |  | Reference |  |
| *≤60 min* | 1.05 (0.78 to 1.41) | .723 | 0.67 (0.50 to 0.91) | .010 |
| *>60 min* | 0.95 (0.67 to 1.37) | .818 | 0.43 (0.29 to 0.66) | < .001 |
| **Chronic disease comorbidity** |  |  |  |  |
| *0* | Reference |  | Reference |  |
| *1-2* | 1.16 (0.89 to 1.53) | .257 | 0.94 (0.70 to 1.27) | .698 |
| *≥3* | 1.35 (0.72 to 2.54) | .341 | 0.93 (0.41 to 2.11) | .857 |
| **Depressive symptoms** |  |  |  |  |
| *No* | Reference |  | Reference |  |
| *Yes* | 1.29 (0.98 to 1.70) | .063 | 1.47 (1.10 to 1.96) | .009 |

Note: Group 1 indicates the slight decline trajectory; Group 2, the sharp drop trajectory; and Group 3, the rise–then–fall trajectory.

Table S8. Baseline sociodemographic characteristics of participants included and not included in the analytic sample

| Characteristics | All (%) | Not included  (*n* = 15,638) | Included  (*n* = 2,055) | *P* |
| --- | --- | --- | --- | --- |
| **Sex** |  |  |  | .282 |
| *Male* | 8,471 (47.88) | 7,510 (48.02) | 961 (46.76) |  |
| *Female* | 9,222 (52.12) | 8,128 (51.98) | 1,094 (53.24) |  |
| **Age** ^a^ | 58 (51, 65) | 58 (51, 66) | 57 (51, 63) | < .001 |
| **Place of residence** |  |  |  | < .001 |
| *Village* | 10,537 (59.50) | 9,215 (58.87) | 1,322 (64.33) |  |
| *City/Town* | 7,171 (40.50) | 6,438 (41.13) | 733 (35.67) |  |
| **Education** |  |  |  | .640 |
| *Less than middle school* | 11,755 (66.59) | 10,396 (66.65) | 1,359 (66.13) |  |
| *Middle school and above* | 5,898 (33.41) | 5,202 (33.35) | 696 (33.87) |  |
| **Marital status** |  |  |  | < .001 |
| *Single* | 2,258 (12.78) | 2,076 (13.29) | 182 (8.86) |  |
| *Married* | 15,417 (87.22) | 13,544 (86.71) | 1,873 (91.14) |  |
| **Smoking status** |  |  |  | .853 |
| *Former* | 1,440 (8.52) | 1,259 (8.48) | 181 (8.81) |  |
| *Never* | 10,596 (62.67) | 9,317 (62.73) | 1,279 (62.24) |  |
| *Now* | 4,871 (28.81) | 4,276 (28.79) | 595 (28.95) |  |
| **Drink status** |  |  |  | .260 |
| *No* | 11,785 (67.14) | 10,435 (67.34) | 1,350 (65.69) |  |
| *<once per month* | 1,384 (7.89) | 1,222 (7.89) | 162 (7.88) |  |
| *≥once per month* | 4,383 (24.97) | 3,840 (24.78) | 543 (26.42) |  |
| **Sleep duration** |  |  |  | .002 |
| *<7 h* | 8,105 (49.92) | 7,044 (49.67) | 1,061 (51.63) |  |
| *7-9 h* | 7,264 (44.74) | 6,347 (44.76) | 917 (44.62) |  |
| *>9 h* | 867 (5.34) | 790 (5.57) | 77 (3.75) |  |
| **Nap duration** |  |  |  | .057 |
| *0 min* | 7,596 (46.79) | 6,637 (46.81) | 959 (46.67) |  |
| *≤60 min* | 6,316 (38.90) | 5,482 (38.66) | 834 (40.58) |  |
| *>60 min* | 2,323 (14.31) | 2,061 (14.53) | 262 (12.75) |  |
| **Chronic disease comorbidity** |  |  |  | < .001 |
| *0* | 5,822 (33.13) | 5,255 (33.86) | 567 (27.59) |  |
| *1-2* | 8,715 (49.59) | 7,649 (49.28) | 1,066 (51.87 |  |
| *≥3* | 3,038 (17.29) | 2,616 (16.86) | 422 (20.54) |  |
| **Depressive symptoms** |  |  |  | .006 |
| *No* | 11,533 (71.91) | 10,108 (72.29) | 1,425 (69.38) |  |
| *Yes* | 4,504 (28.09) | 3,875 (27.71) | 629 (30.62) |  |

Note: ^a^: Age is presented as median (interquartile range) because of non-normal distribution. The differences in baseline age between participants included in the analytic sample and those excluded were assessed using the Mann–Whitney U test. Missing data were as follows — education: 55 in the excluded group; marital status: 33 in the excluded group; smoking: 801 in the excluded group; sleep duration: 1,472 in the excluded group; nap duration: 1,473 in the excluded group; chronic diseases: 133 in the excluded group; depressive symptoms: 1,670 in the excluded group and 1 in the included group.

Table S9. Sex‐stratified associations between physical activity trajectories and cognitive function

|  | Male  *β* (95% CI) | *P* | Female  *β* (95% CI) | *P* |
| --- | --- | --- | --- | --- |
| **Total cognitive function score** | | | | |
| *Group 1* | Reference |  | Reference |  |
| *Group 2* | –0.079 (–0.594 to 0.435) | .763 | –0.577 (–1.141 to –0.011) | **.045** |
| *Group 3* | –0.563 (–1.102 to –0.025) | **.040** | –0.707 (–1.308 to –0.106) | **.021** |
| **Episodic memory score** | | | | |
| *Group 1* | Reference |  | Reference |  |
| *Group 2* | 0.013 (–0.353 to 0.379) | .944 | –0.381 (–0.777 to 0.014) | .059 |
| *Group 3* | –0.183 (–0.567 to 0.199) | .347 | –0.178 (–0.600 to 0.242) | .405 |
| **Mental state score** | | | | |
| *Group 1* | Reference |  | Reference |  |
| *Group 2* | –0.092 (–0.341 to 0.157) | .468 | –0.195 (–0.481 to 0.091) | .182 |
| *Group 3* | –0.380 (–0.641 to –0.119) | **.004** | –0.528 (–0.833 to –0.223) | **.001** |

Note: The model was adjusted for age, place of residence, education level, marital status, smoking status, drinking status, sleep duration, nap duration, number of chronic diseases, and depressive symptoms. Bolded p-values indicate statistical significance at *P* < .05. Group 1 indicates the slight decline trajectory; Group 2, the sharp drop trajectory; and Group 3, the rise–then–fall trajectory.

Table S10. Age‐stratified associations between physical activity trajectories and cognitive function

|  | 54-59  *β* (95% CI) | *P* | 60-69  *β* (95% CI) | *P* | 70-101  *β* (95% CI) | *P* |
| --- | --- | --- | --- | --- | --- | --- |
| **Total cognitive function score** | | | | | | |
| *Group 1* | Reference |  | Reference |  | Reference |  |
| *Group 2* | –0.267 (–0.949 to 0.415) | .443 | –0.490 (–1.050 to 0.068) | .085 | –0.084 (–0.840 to 0.670) | .825 |
| *Group 3* | –0.829 (–1.514 to –0.144) | **.018** | –0.645 (–1.181 to –0.109) | **.018** | –0.490 (–1.472 to 0.490) | .327 |
| **Episodic memory score** | | | | | | |
| *Group 1* | Reference |  | Reference |  | Reference |  |
| *Group 2* | –0.166 (–0.652 to 0.319) | .501 | –0.259 (–0.667 to 0.148) | .212 | –0.021 (–0.538 to 0.494) | .934 |
| *Group 3* | –0.365 (–0.853 to 0.121) | .141 | –0.274 (–0.665 to 0.115) | .168 | 0.136 (–0.535 to 0.807) | .691 |
| **Mental state score** | | | | | | |
| *Group 1* | Reference |  | Reference |  | Reference |  |
| *Group 2* | –0.100 (–0.451 to 0.250) | .574 | –0.231 (–0.512 to 0.050) | .108 | –0.063 (–0.429 to 0.303) | .736 |
| *Group 3* | –0.463 (–0.815 to –0.111) | **.010** | –0.370 (–0.640 to –0.100) | **.007** | –0.626 (–1.102 to –0.151) | **.010** |

Note: The model was adjusted for sex, place of residence, education level, marital status, smoking status, drinking status, sleep duration, nap duration, number of chronic diseases, and depressive symptoms. Bolded *P*-values indicate statistical significance at *P* < .05. Group 1 indicates the slight decline trajectory; Group 2, the sharp drop trajectory; and Group 3, the rise–then–fall trajectory.

Table S11. Education‐stratified associations between physical activity trajectories and cognitive function

|  | Less than middle school  *β* (95% CI) | *P* | Middle school and above  *β* (95% CI) | *P* |
| --- | --- | --- | --- | --- |
| **Total cognitive function score** | | | | |
| *Group 1* | Reference |  | Reference |  |
| *Group 2* | –0.351 (–0.844 to 0.141) | .162 | –0.067 (–0.639 to 0.504) | .816 |
| *Group 3* | –0.501 (–1.039 to 0.037) | .068 | –0.869 (–1.418 to –0.319) | **.002** |
| **Episodic memory score** | | | | |
| *Group 1* | Reference |  | Reference |  |
| *Group 2* | –0.225 (–0.567 to 0.116) | .196 | 0.091 (–0.340 to 0.523) | .677 |
| *Group 3* | –0.063 (–0.437 to 0.309) | .738 | –0.400 (–0.815 to 0.013) | .058 |
| **Mental state score** | | | | |
| *Group 1* | Reference |  | Reference |  |
| *Group 2* | –0.126 (–0.372 to 0.120) | .316 | –0.159 (–0.437 to 0.117) | .259 |
| *Group 3* | –0.437 (–0.706 to –0.168) | **.001** | –0.468 (–0.734 to –0.201) | **.001** |

Note: The model was adjusted for sex, age, place of residence, marital status, smoking status, drinking status, sleep duration, nap duration, number of chronic diseases, and depressive symptoms. Bolded *P*-values indicate statistical significance at *P* < .05. Group 1 indicates the slight decline trajectory; Group 2, the sharp drop trajectory; and Group 3, the rise–then–fall trajectory.

Table S12. Multivariable linear regression of physical activity trajectories on cognitive scores using truncated IPTW (1st–99th percentiles)

|  | Total cognitive function score | | Episodic memory score | | Mental state score | |
| --- | --- | --- | --- | --- | --- | --- |
|  | Truncated IPTW  *β* (95% CI) | *P* | Truncated IPTW  *β* (95% CI) | *P* | Truncated IPTW  *β* (95% CI) | *P* |
| Group 1 | Reference |  | Reference |  | Reference |  |
| Group 2 | –0.385 (–0.810 to 0.040) | .076 | –0.170 (–0.452 to 0.112) | .237 | –0.215 (–0.438 to 0.008) | .059 |
| Group 3 | –0.718 (–1.157 to –0.280) | .001 | –0.192 (–0.504 to 0.118) | .225 | –0.525 (–0.746 to –0.305) | < .001 |

Note: IPTW: inverse probability of treatment weighting. Models were adjusted for sex, age, residence, education level, marital status, smoking status, drinking status, sleep duration, nap duration, number of chronic diseases, and depressive symptoms. Group 1 indicates the slight decline trajectory; Group 2, the sharp drop trajectory; and Group 3, the rise–then–fall trajectory.

Table S13. E values for the association between physical activity trajectories on cognitive scores

| Variables | E-Value (CI Limit) | | |
| --- | --- | --- | --- |
|  | Model 1 | Model 2 | Model 3 |
| **Total cognitive function score** | | | |
| *Group 2* ***vs*** *Group 1* | 1.375 (1.000) | 1.412 (1.000) | 1.370 (1.000) |
| *Group 3* ***vs*** *Group 1* | 1.648 (1.324) | 1.690 (1.369) | 1.638 (1.313) |
| **Episodic memory score** | | | |
| *Group 2* ***vs*** *Group 1* | 1.310 (1.000) | 1.341 (1.000) | 1.306 (1.000) |
| *Group 3* ***vs*** *Group 1* | 1.343 (1.000) | 1.391 (1.000) | 1.340 (1.000) |
| **Mental state score** | | | |
| *Group 2* ***vs*** *Group 1* | 1.375 (1.000) | 1.410 (1.000) | 1.373 (1.000) |
| *Group 3* ***vs*** *Group 1* | 1.887 (1.572) | 1.917 (1.598) | 1.871 (1.556) |

Note: Model 1: adjusted for sex, age, residence, education level, and marital status. Model 2: added smoking status, drinking status, sleep duration, and nap duration to model 1. Model 3: added number of chronic diseases and depressive symptoms to model 2. CI Limit refers to the lower confidence interval bound of the E-value, indicating the minimum strength of association that an unmeasured confounder would need to fully explain away the observed effect. Group 1 indicates the slight decline trajectory; Group 2, the sharp drop trajectory; and Group 3, the rise–then–fall trajectory.

Table S14. Sensitivity analysis of the associations between physical activity trajectories and cognitive outcomes after additional adjustment for baseline cognitive function

|  | Before additional adjustment for baseline cognitive function | | After additional adjustment for baseline cognitive function | |
| --- | --- | --- | --- | --- |
|  | OR/*β*(95% CI) | *P* | OR/*β*(95% CI) | *P* |
| **Cognitive impairment** | | | | |
| Group 1 | Reference |  | Reference |  |
| Group 2 | 1.36 (0.94 to 1.96) | .098 | 1.27 (0.87 to 1.86) | .204 |
| Group 3 | 1.35 (0.90 to 2.04) | .145 | 1.27 (0.83 to 1.94) | .257 |
| **Total cognitive function score** | | | | |
| Group 1 | Reference |  | Reference |  |
| Group 2 | –0.286 (–0.669 to 0.095) | .141 | –0.160 (–0.516 to 0.196) | .377 |
| Group 3 | –0.621 (–1.022 to –0.219) | .002 | –0.448 (–0.823 to –0.074) | .019 |
| **Episodic memory score** | | | | |
| Group 1 | Reference |  | Reference |  |
| Group 2 | –0.145 (–0.415 to 0.123) | .288 | –0.076 (–0.335 to 0.182) | .562 |
| Group 3 | –0.171 (–0.455 to 0.111) | .234 | –0.076 (–0.349 to 0.194) | .576 |
| **Mental state score** | | | | |
| Group 1 | Reference |  | Reference |  |
| Group 2 | –0.141 (–0.331 to 0.049) | .145 | –0.083 (–0.263 to 0.095) | .359 |
| Group 3 | –0.449 (–0.648 to –0.249) | < .001 | –0.371 (–0.559 to –0.182) | < .001 |

Note: *β* values were obtained from multivariable linear regression models for continuous cognitive scores, and OR values were obtained from multivariable logistic regression models for cognitive impairment. The models before additional adjustment for baseline cognitive function were adjusted for sex, age, residence, education level, marital status, smoking status, drinking status, sleep duration, nap duration, number of chronic diseases, and depressive symptoms. The models after additional adjustment further included baseline cognitive function. Group 1 indicates the slight decline trajectory; Group 2, the sharp drop trajectory; and Group 3, the rise–then–fall trajectory.

**Reference**

1.	Nagin DS, Odgers CL. Group-based trajectory modeling in clinical research. Annu Rev Clin Psychol. 2010;6:109-38.

2.	VanderWeele TJ, Ding P. Sensitivity Analysis in Observational Research: Introducing the E-Value. Ann Intern Med. 2017;167(4):268-74.
